# Improving anthelmintic treatment for schistosomiasis and soil-transmitted helminthiases through sharing and reuse of individual participant data

**DOI:** 10.12688/wellcomeopenres.17468.1

**Published:** 2022-01-05

**Authors:** Martin Walker, Luzia T. Freitas, Julia B. Halder, Matthew Brack, Jennifer Keiser, Charles H. King, Bruno Levecke, Yvonne Ai-Lian Lim, Otavio Pieri, Doudou Sow, J. Russell Stothard, Joanne P. Webster, Xiao-Nong Zhou, Robert F. Terry, Philippe J. Guérin, Maria-Gloria Basáñez

**Affiliations:** 1 Department of Pathobiology and Population Sciences, Royal Veterinary College, London, Hatfield, UK; 2 Department of Infectious Disease Epidemiology, MRC Centre for Global Infectious Disease Analysis and London Centre for Neglected Tropical Disease Research, Imperial College London, London, UK; 3 Infectious Diseases Data Observatory, University of Oxford, Oxford, UK; 4 Swiss Tropical and Public Health Institute, Basel, Switzerland; 5 University of Basel, Basel, Switzerland; 6 Center for Global Health and Diseases, Case Western Reserve University, Cleveland, Ohio, USA; 7 Department of Translational Physiology, Ghent University, Merelbeke, Belgium; 8 Department of Parasitology, University of Malaya, Kuala Lumpur, Malaysia; 9 Laboratory of Health and Environment Education, Oswaldo Cruz Institute, Fiocruz, Rio de Janiero, Brazil; 10 Service de Parasitologie, Université Gaston Berger de Saint Louis, Saint Louis, Senegal; 11 Department of Tropical Disease Biology, Liverpool School of Tropical Medicine, Liverpool, UK; 12 National Institute of Parasitic Diseases, China Center for Disease Control and Prevention, Shanghai, China; 13 Special Programme for Research and Training in Tropical Diseases (TDR), World Health Organization, Geneva, Switzerland

**Keywords:** schistosomiasis, soil-transmitted helminthiasis, data sharing, data reuse, treatment, anthelmintic, neglected tropical diseases

## Abstract

The Infectious Diseases Data Observatory (IDDO, https://www.iddo.org) has launched a clinical data platform for the collation, curation, standardisation and reuse of individual participant data (IPD) on treatments for two of the most globally important neglected tropical diseases (NTDs), schistosomiasis (SCH) and soil-transmitted helminthiases (STHs). This initiative aims to harness the power of data-sharing by facilitating collaborative joint analyses of pooled datasets to generate robust evidence on the efficacy and safety of anthelminthic treatment regimens. A crucial component of this endeavour has been the development of a Research Agenda to promote engagement with the SCH and STH research and disease control communities by highlighting key questions that could be tackled using data shared through the IDDO platform. Here, we give a contextual overview of the priority research themes articulated in the Research Agenda—a ‘living’ document hosted on the IDDO website—and describe the three-stage consultation process behind its development. We also discuss the sustainability and future directions of the platform, emphasising throughout the power and promise of ethical and equitable sharing and reuse of clinical data to support the elimination of NTDs.

## Disclaimer

The views expressed in this article are those of the authors. Publication in Wellcome Open Research does not imply endorsement by Wellcome.

## Introduction

At least one billion of the world’s poorest people suffer from neglected tropical diseases (NTDs). Two of the most common NTDs are schistosomiasis (SCH) and soil-transmitted helminthiases (STHs), caused by parasitic worms (helminths, trematodes and nematodes, respectively) that are endemic throughout the tropical and sub-tropical regions and intimately associated with poverty ^1– 3^. The World Health Organization (WHO), supported by global health partners, has spearheaded efforts to eliminate these diseases as a public health problem by 2030 ^4^, predominantly using a strategy of preventive chemotherapy (PC). This entails the distribution of anthelmintic drugs (anthelmintics) to at-risk populations on an annual or semi-annual basis, by mass drug administration (MDA), upon pre-determined infection prevalence thresholds.

The scale up of these PC programs over the past decade is unprecedented and they are among the first public health interventions to resume after recent disruptions caused by the coronavirus disease 2019 (COVID-19) pandemic. Every year, since 2017, more than a billion people have been treated for NTDs ^5, 6^, including in 2019, 105 million people were given praziquantel for SCH and 613 million people were given benzimidazoles for STHs ^7^. Despite this, there remain questions on the factors that shape individual responses to treatment; responses to treatment in understudied groups; anthelmintic safety and tolerability profiles, and methodological questions on how and when responses should be measured and how future studies, including clinical trials, should be best designed to address these questions. Moreover, although the spectre of emerging anthelmintic resistance is ever-present—having already arisen to all major classes of anthelmintics in the veterinary field ^8– 11^—there remains little systematic monitoring of anthelmintic efficacy in human populations.

The Infectious Diseases Data Observatory (IDDO) is working with the SCH and STH research and disease control communities to develop a clinical data platform for the collation, curation, standardisation and reuse of individual participant data (IPD) on treatment responses to anthelmintics. It is becoming increasingly well-recognised that data sharing and curation to a standardised format maximises the utility of data by enabling joint analysis of pooled datasets to increase the power of analysis, uncover new information and generate new evidence ^12– 14^. The goal of the IDDO initiative is to facilitate collaborative research to improve the efficacy and sustainability of treatments for SCH and STHs through ethical and equitable sharing and reuse of data for scientific outputs that produce better evidence ^13, 15, 16^.

As part of the platform development phase, we have developed a Research Agenda highlighting research questions that could be tackled using data shared through the IDDO platform. This is intended to spur the SCH and STHs research communities to engage with the platform which will ultimately enhance research aimed at improving treatments for these diseases. Here, we first describe the consultative process used to develop the Research Agenda, an approach shared across the IDDO portfolio of infectious diseases. We then provide a contextual overview of the priority research areas identified by this process, highlighting areas where sufficient data exist and could be tackled within the short- to mid-term (2–3 years) as well as questions for which there are currently insufficient data, or which are out of the current scope of the platform, but which may in the long-term be important or trigger new research.

## Developing the Research Agenda

By taking an inclusive, consultative approach to developing the Research Agenda we have promoted engagement with the SCH and STHs research and disease control communities and developed a document to spur new research. In Box 1, we describe briefly how the Research Agenda developed for visceral leishmaniasis (VL)—another NTD in the IDDO portfolio—has successfully galvanised the VL research community.

**Box 1. Development of a Research Agenda for visceral leishmaniasis**

**Figure 1.**
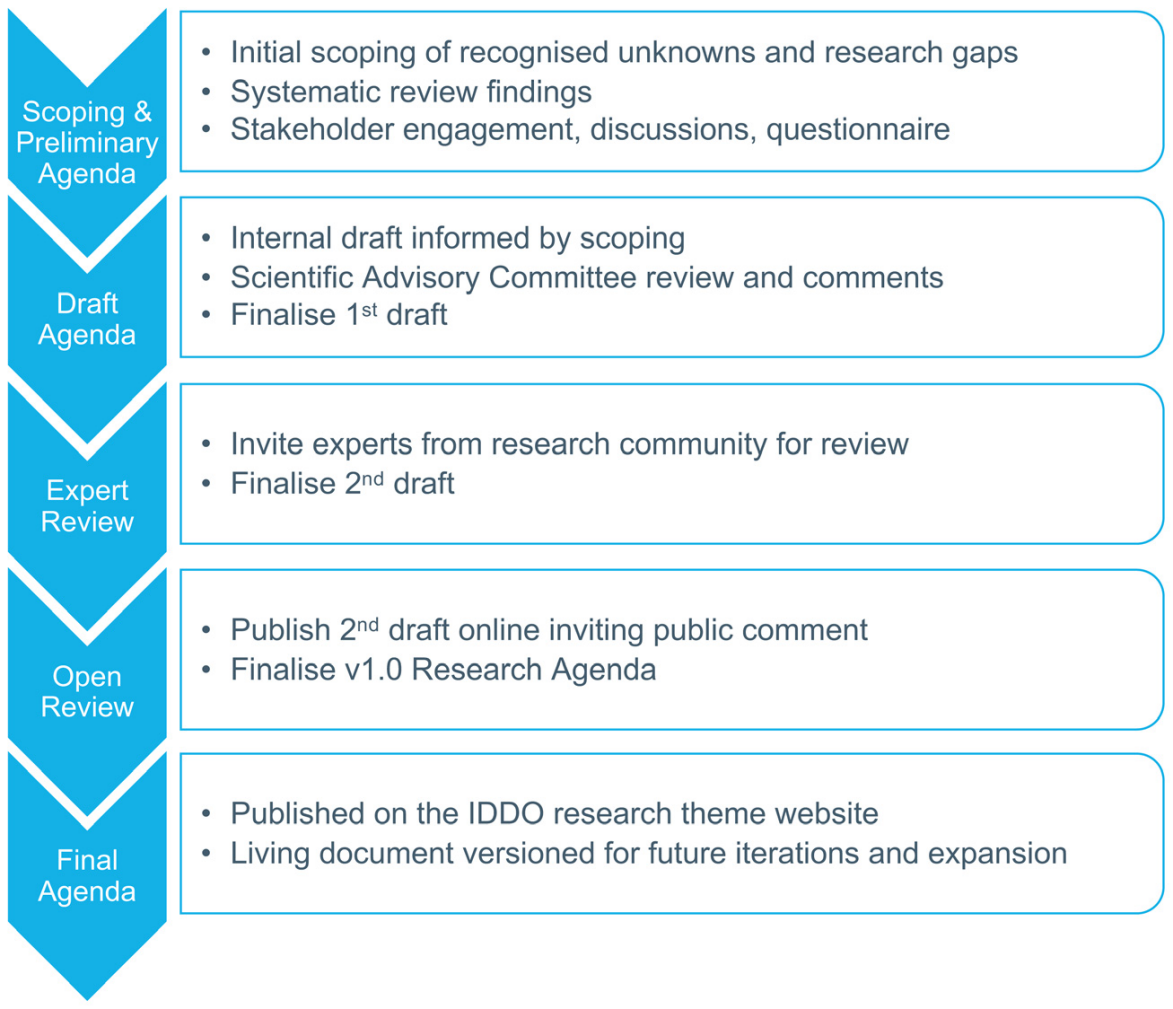
The Infectious Diseases Data Observatory (IDDO) Research Agenda development process. Schematic reproduced with permission from https://www.iddo.org/research-agenda-development-process.

The Research Agenda for visceral leishmaniasis (VL) was completed in 2019 following the same consultative development process as used for the schistosomiasis and soil-transmitted helminthiases Research Agenda as outlined in Figure 1. The VL Research Agenda provided a clear indication of which knowledge gaps could be addressed by the research community if the individual participant data (IPD) were made available. This inspired research proposals for two collaborative Study Groups which were drafted with the VL Scientific Advisory Committee, as representatives of the global VL research community, and key partners, including non-governmental organisations, regional health agencies and funders dedicated to serving VL patients. These proposals were in turn shared with relevant investigators identified from the scoping review inviting them to contribute data and participate in the analysis for each Study Group. By presenting clear research proposals to investigators outlining the outcomes to be achieved through their data contribution, these Study Groups—which were originally inspired by the Research Agenda—have greatly enhanced data contribution, participation and engagement with the VL platform and have led to collaborative analyses of data received from across all endemic regions.

The SCH and STH Research Agenda has been developed following the same process as used for VL (and across IDDO platforms, Figure 1), reflecting topics identified by systematic scoping reviews ^17, 18^, engagement with the communities, and written and revised through a three-stage consultation process:

1. Internal process: development of the first draft by the Secretariat and the Scientific Advisory Committee (SAC) members
2. External expert process: review of the first draft by key experts in the field and production of a second version of the research agenda
3. Public consultation process: the second version is publicly shared with the scientific community and comments were called from April to July 2020 to produce a first ‘living document’ that can be updated as the research needs of the disease communities evolve.

Version 1.0 of the SCH and STHs Research Agenda is hosted on the IDDO website. This document reflects current consensus from the scientific community on priority research areas that could help to improve treatment (and control/elimination) of SCH and STHs. Hereafter, we provide a contextual overview of these research areas.

## Heterogeneity in treatment responses

Responses to anthelmintic treatments vary among individuals ^19– 22^ and among populations ^23, 24^. Variation in treatment responses can be driven by multiple factors, including those relating to demography, spatiotemporal effects ^22, 23, 25, 26^, such as potentially emerging anthelminthic resistance or the distribution of low-quality medicine ^27, 28^, drug effects, such as differences in the efficacy of treatment regimens and poorly understood individual-level differences. Heterogeneity is also enhanced by methodological, study design and reporting variation that affect the apparent treatment response (i.e., the response measured through the lens of a particular diagnostic used in a particular manner and reported in a particular way). Indeed, it is because of these high levels of heterogeneity in study methodologies, analyses and reporting ^17, 18^ that discriminating and quantifying different sources of variation using only group/population-level responses (i.e. traditional meta-analyses) becomes difficult to impossible. Comprehensive IPD meta-analyses can overcome some of these challenges and would permit better understanding of the role of different pharmacological, (host and parasite) biological and methodological components in shaping treatment responses. In turn, such understanding will enable optimal evidence-based recommendations and guidance on how best to deploy the medicines to current and future demographic groups that are the cornerstone of global efforts to control and eliminate SCH and STHs.

### Characterising spatiotemporal heterogeneity

For both SCH and STHs, there exist examples of geographical and temporal variability in responses to anthelminthics, which have sometimes been linked to the duration of MDA, a proxy of drug pressure and potential driver of anthelminthic resistance. In Uganda, the efficacy of praziquantel against intestinal SCH caused by *Schistosoma mansoni*, has been shown to be lower in schools with a longer duration (higher drug pressure) of MDA ^29^. Elsewhere, *S. mansoni* populations with reduced susceptibility to praziquantel have been documented in Egypt ^30^, Kenya ^31^ and Senegal ^32^. Notwithstanding these reports—and although resistance to praziquantel can be induced in the laboratory—and can emerge in natural populations ^30^, there remains no conclusive evidence for its establishment in the field and the topic remains controversial ^25, 33, 34^. Moreover, a recent meta-analysis found that the efficacy of praziquantel has been maintained since its introduction as the preferred treatment for SCH in the late 1970s ^35^.

Links between the duration of MDA and the efficacy of benzimidazoles have also been reported. In Pemba Island, which has a long history of MDA, poor efficacy of albendazole against *Trichuris trichiura* (whipworm) and hookworm has been reported ^22, 23, 36^, and a population-level meta-analysis found the efficacy of benzimidazoles against whipworm had declined globally between 1995 and 2015 (although this global trend may be biased by the abundance of trials conducted in Pemba Island where the intensity of whipworm is very high and responses are consistently poor) ^26^. Benzimidazole treatment is known to select for *β*-tubulin mutations which are associated with resistance ^37, 38^ (and particularly so in helminths of veterinary importance ^39^) although currently, no direct association between duration of MDA (drug pressure) and the selection of *β*-tubulin mutations resulting in reduced efficacy has been documented in the species infecting human populations.

Naturally, factors other than emerging resistance can shape treatment efficacy variation over time and space. An important neglected possible driver is medicine quality, which may differ between countries and regions depending on the source and distribution of the drugs. For example, a relatively high prevalence of poor quality benzimidazoles—linked to the country of origin—has been identified in Ethiopia ^28^ and quality has also shown to be variable among brands and could cause variable responses ^27^. The paucity of published studies that explore medicine quality for SCH ^40^ and STHs ^27, 28^ means that it is unclear how quality may shape spatiotemporal heterogeneity in responses. In malaria, poor-quality medicines are recognised as a determinant of treatment failure, morbidity, mortality and drug resistance ^41– 43^. The IDDO Medicine Quality Research Group is dedicated to strengthening knowledge about the scale and extent of problems associated with substandard and falsified medicines for human and veterinary diseases.

Numerous factors such as co-infections, drug-drug and host-drug interactions may also shape the response landscape. Many of these may be difficult to distinguish from the variables that typically comprise clinical data. Nevertheless, there exist some intriguing possibilities such as genetic diversity in the cytochrome P _450_ enzyme across SCH-endemic regions in Africa being linked to the metabolism—and possibly the efficacy—of praziquantel ^44, 45^. But ultimately, a better phenomenological understanding of treatment efficacy—irrespective of the underlying drivers—would be highly beneficial from a pragmatic perspective, particularly as this is so crucial to the effectiveness of PC programmes.

Consequently, we envisage that an important first step in better characterising spatiotemporal heterogeneity will be to map and visualise anthelminthic responses ( Table 1), inspired by the pioneering work of the Worldwide Antimalarial Resistance Network (WWARN) in tracking the emergence and spread of artemisinin-resistant *Plasmodium falciparum* malaria in Southeast Asia ^46^. This would provide an overview of the current geographical picture of anthelmintic responses, and spur more detailed analysis of the IPD hosted by the platform to identify drivers of geographical and temporal heterogeneity, particularly with respect to PC history. This process can begin now with existing submissions to the IDDO platform and be updated as new data are contributed.

**Table 1.** Priority research themes, activities & timescales.

Research theme	Activity	Timescale
Characterising spatiotemporal variation in treatment responses	• Development of an efficacy explorer to map responses in space and time • Engagement to increase research and data collection on anthelminthic quality	Short-term Mid-/long-term
Improving evidence base for treatment regimens	• Individual participant data (IPD) meta-analyses on drug combinations for treating hookworm & *Trichuris trichiura* (whipworm) • IPD meta-analyses on effect of dose and frequency of administration on efficacy of praziquantel (e.g. 40 mg/kg vs. 60 mg/kg in single or multiple doses)	Short-/mid-term Short-/mid-term
Evaluating new antigen- detection & molecular diagnostics	• Define reference range of responses to praziquantel measured using antigen detection methods (CCA or CAA) • Comparison of assays and laboratory protocols for molecular identification of soil-transmitted helminth infection and promotion of standardised approaches	Short-/mid-term Short-/mid-term
Standardising study design and reporting	• Development of a case record form incorporating the data standard developed by the Clinical Data Interchange Standards Consortium (CDISC) • Engagement to foster adoption of standardised protocols and reporting	Short-term Mid-/long-term
Medicine safety in pregnancy	• IPD meta-analyses of safety (and efficacy) of praziquantel and benzimidazoles in pregnant and breastfeeding women	Short-/mid-term
Medicine formulation for preschool-age children	• Collation, curation & standardisation of phase III clinical trial data on paediatric praziquantel formulation	Short-/mid-term
Analytical approaches	• Testing, comparison and recommendations on optimal statistical approaches for IPD (meta-) analyses using ‘gold standard’ datasets	Short-/mid-term

### Improving evidence-base for treatment regimens

Praziquantel is used exclusively for PC regimens against SCH, typically given at a dose of 40 mg/kg. Some (but not all ^47^) studies have shown 60 mg/kg (typically divided into three 20 mg/kg doses given over the course of a single day) to be more efficacious than the single 40 mg/kg dose ^19, 35, 48, 49^. There also exists evidence that repeated doses of praziquantel may improve responses ^50^ and that co-administration of food increases bioavailability ^51, 52^, which is increasingly recommended as best practice in MDA. Moreover, the efficacy of praziquantel is variable against different *Schistosoma* species ^53^—with little yet known on the susceptibility of hybrids ^54– 56^—indicating that modified regimens could be recommended where either intestinal or urogenital infections dominate.

Preventive chemotherapy programmes for STHs are based on single-dose benzimidazoles (albendazole or mebendazole). However, this regimen has poor efficacy against *T. trichiura* ^26, 57, 58^, particularly in heavily infected individuals ^59, 60^, and the efficacy of mebendazole against hookworm has also been questioned ^61, 62^. There is thus growing consensus that more efficacious regimens are needed to reach control and elimination goals ^22^. Combination therapies of benzimidazoles with ivermectin ^63– 66^, oxantel pamoate ^63, 67, 68^ or moxidectin ^69^, and tribendimidine with oxantel pamoate ^67^ have shown substantive improvement in efficacy compared to benzimidazoles alone ^57^.

 Other demographic (e.g. age, sex), socioeconomic, nutritional and health indicators may influence how an individual responds to treatment. For example, treatment efficacy may be affected by the intensity of both schistosome ^70– 72^ and soil-transmitted helminths ^24, 59, 60, 73^ infections, and responses may vary among age groups (e.g. 19, 24). Combined analyses of existing IPD could provide the strength of evidence required to prompt revised recommendations on optimised PC, critically in terms of optimal dosing to treat SCH and optimal combinations to treat STHs ( Table 1), but also considering other individual-level factors that may be important determinants of treatment response. A more nuanced approach to treatment recommendations may be required to take into consideration the various factors that can modify treatment responses, including the geographical distribution and local dominance of different schistosome and soil-transmitted helminth infections.

### Evaluating new antigen-detection & molecular diagnostics

Traditionally, responses to anthelmintic treatment have been measured using classical microscopy-based parasitological techniques. Indeed, the WHO defines responses as either ‘satisfactory’, ‘doubtful’ or ‘reduced’ based on so-called egg reduction rates (ERRs), the percent reduction in the post-treatment parasitological egg count compared to the corresponding pre-treatment measurement in a population of treated individuals ^74^. However, new antigen-detection and molecular techniques—such as the detection of schistosome circulating cathodic or anodic antigen (CCA/CAA) in urine or serum ^75, 76^ and the quantification of STH DNA by PCR of stool samples ^23, 77, 78^—are increasingly being used as more sensitive alternatives to classical parasitological diagnostic techniques for evaluating treatment responses.

 The move towards molecular approaches (particularly in research contexts) brings challenges when interpreting responses that have for decades been quantified using well-understood parasitological measures ( Table 1). More sensitive molecular and antigen-detection diagnostics may yield estimates of efficacy that are lower than those measured by traditional parasitological methods, but this may also depend on the infecting species, when after treatment assessments are undertaken, and what parasitological technique it is being compared with. For example, CCA/CAA levels drop rapidly (within 24 hours) after treatment of schistosome infection with praziquantel ^79– 81^ but may indicate lower efficacy than parasitology-based assessments (e.g. Kato Katz) because of their higher sensitivity for detecting low-level infections ^82^. This is further complicated by the differential performance of antigen-detection diagnostics for the detection of intestinal and urogenital schistosome infections ^83, 84^.

 The standardisation and commercial availability of some antigen-detection diagnostics (e.g. point-of-care CCA) provide advantages in comparability of results between studies (as well as for other activities such as epidemiological mapping). However, most diagnostics are not standardised and there remain questions on the comparability of molecular diagnostics results derived from assays run in different laboratories ^85– 88^ and different epidemiological settings ^89^. Ultimately, the sharing of data and detailed laboratory protocols and procedures will permit formal comparison of diagnostics both within the context of assessing responses to anthelmintics and more generally ^87, 88^.

### Standardising study design and reporting

The design of a study assessing anthelmintic responses is crucial to the interpretation of the resulting data. For example, it is known that post-treatment egg counts for both schistosome and soil-transmitted helminths infections tend to be at their lowest approximately 2–3 weeks after treatment, which is why this time window is recommended for efficacy assessment ^90, 91^. Although there maybe subtleties in optimal timing for different infections (e.g. between *S. mansoni* ^92^ and *S. haematobium* ^93^), it is more important that a standardised and adequate follow-up time is employed consistently to facilitate comparison and interpretability of results, although the time window for assessment may be quite different for detection of antigens rather than excreted eggs ^79^. Similar arguments can be made for the ubiquitous use of the Kato-Katz method for egg microscopy; any inferiority in performance compared to other diagnostics maybe outweighed by the advantages of standardisation ^23^.

 Notwithstanding, even widely used and relatively standardised tools have elements that can vary between studies and should be recorded (just as detailed protocols should be documented for molecular laboratory techniques). For example, even Kato-Katz test kits from different providers may sometimes yield differences in weight of stool (although this has not been found to require adjustment to the multiplication factor to convert to eggs per gram ^94^). Therefore, details of manufacturers and other specifications of diagnostics and study protocols should be recorded and reported in a standardised fashion to enhance interpretability and comparison. Similarly, there will be variation among technicians in the reading and preparing of Kato-Katz slides, and other microscopy-based approaches, which should be captured by recording individual identifiers for who processed each slide as observed in malaria ^95^.

 Variability in eligibility criteria among studies can also lead to systematic differences and bias in study outcomes. For example, a common inclusion criterion in an efficacy assessment is for individuals to be diagnosed with infection using a single Kato-Katz slide. But this can lead to positive bias (overestimation) of drug efficacy, particularly when infection levels and/or efficacy are/is low. This can be mitigated by re-testing individuals after the initial eligibility screen and using this re-test measure of infection in subsequent estimations of drug efficacy ^96^.

The IDDO platform intends to facilitate the development of a standard case reporting form (CRF) to foster increased standardisation of protocols and reporting for clinical studies on SCH and STHs ( Table 1). This will be achieved through engagement with the respective research communities and will be informed by current and future contributions to the platform. The CRF will also integrate the principles of the Clinical Data Interchange Standards Consortium (CDISC) clinical data standards that the platform is using to curate data contributions, further enhancing data interpretability, clarity and interoperability.

## Medicine safety, tolerability and side effects

The drugs used to treat SCH and STHs are considered very safe and associated predominantly with only relatively mild side effects, although the published research in this area is limited. A particular strength of aggregating IPD from multiple studies is to increase power when individual studies are comparatively scarce. Side effects of praziquantel, particularly in heavily infected individuals, can be substantial ^97, 98^ and may have a deleterious effect on participation with subsequent rounds of MDA ^99^. Side effects of benzimidazoles are generally mild (but see 100), including in young children under 5 years old ^101, 102^ but it will be necessary to assess side effect and tolerability profiles of new combination therapies that improve treatment of *T. trichiura* and hookworm infections ^63, 103^. A key component of any analysis of tolerability is the robust assessment of bias; studies actively collecting information on adverse events are likely rare and those based on passive surveillance, such as during MDA programmes, will be subject to reporting bias ^104^.

## Safety and responses in understudied groups

Determining safety and drug responses in understudied groups is crucial to defining appropriate inclusion/exclusion criteria for PC. This directly impacts the success of PC programmes that must strive to maximise therapeutic coverage to meet control and elimination goals. Like studies on side effects, data on understudied groups are, by definition, limited which only emphasises further the importance of data sharing to maximise the information and power of IPD. Indeed, the ‘leaving no one behind’ principle, as articulated in the Sustainable Development Goals, has gained prominence as an indicator of neglected tropical disease (NTD) programme success ^105^.

### Medicine safety in pregnancy

The WHO recommend inclusion of pregnant and breastfeeding women for treatment with praziquantel based on studies that have indicated that it is safe and efficacious ^106– 108^. However, praziquantel is still frequently not offered during PC to pregnant or breastfeeding women, sometimes because of prioritisation of school-based rather than community-based delivery of MDA ^109^. Data on the safety of other anthelmintics for use in pregnancy are limited ^110, 111^ and current recommendations indicate treatment only on the second and third trimesters as an important component of antenatal care ^112, 113^. New updated analyses using pooled IPD could add considerable value to the evidence-base to support treatment recommendations ( Table 1).

### Medicine formulations for preschool-age children

In recent years it has been recognised that treating preschool-age children (less than 5 years old) for SCH may have substantial benefits for preventing morbidity and reducing community transmission ^45, 114^. It is recommended that children aged ≤5 years be given a 40 mg/kg dose of praziquantel (doses above 40 mg/kg appear to offer no improvement in response) ^47, 115^ as crushed tablets ^45^. However, the bitter taste of praziquantel has led to operational difficulties ^47, 116^ and spurred the development of a paediatric formulation by the Paediatric Praziquantel Consortium. Phase III trials are currently ongoing and an important component along the pathway towards registration will be the curation and standardisation of the clinical data and, ideally, its sharing for scrutiny and comparison with existing formulations ( Table 1). More research should also be conducted on the pharmacokinetics of praziquantel in preschool-age children and its bioavailability profile ^45^.

 The optimal formulation of benzimidazoles for STHs, which are periodically given to preschool-age children over 12 months old ^101^, also remains an open question. Both albendazole and mebendazole are available as chewable tablets ^117– 119^ or can be crushed ^120^ to minimise the likelihood of choking events ^100, 120^. Collating available safety data on the administration of benzimidazole tablets to preschool-age children could provide important information on the frequency of these adverse events and, crucially, highlight safest modes of administration.

## Analytical approaches

The WHO provides guidance on analysis protocols for calculating anthelmintic efficacy as ERRs on a population-level basis ^90^. These approaches, however, do not extend to IPD when incorporating covariates of the treatment response. A number of new methods have been proposed in both the human ^19– 22, 121^ and veterinary ^122, 123^ domains for analysing IPD, but there remain no standardised or consensus approach and many unresolved technical questions. For example, how quantitative expressions of the distribution of drug responses among individuals (e.g., percentage of individuals with a ‘satisfactory’ or ‘reduced’ response) relate to more traditional population-level summaries (e.g., average ERR or cure rate), how accurately can individual suboptimal (or ‘reduced’) responses be identified, and importantly, be distinguished from response variation not associated with decreased efficacy? should responses measured using (multiple) molecular diagnostics be integrated into analytical frameworks? These questions will not be answered by the sharing of data *per se*, but the assemblage of an abundance of IPD will provide the raw material to test different analytical approaches. We envisage that the platform will play an important role in facilitating collaboration to improve the quality and consistency of methods for IPD meta-analyses in the helminth and NTD domain.

## Conclusions

Here, we have given a contextual overview of the Research Agenda that has been developed as part of the process building a SCH and STHs sharing and reuse platform for clinical data. The Research Agenda is not intended to be either prescriptive or exhaustive, but rather to provide a guide to research questions—identified as priorities by the disease control and research communities—that could be tackled through the sharing and reuse of IPD identified by scoping review. Clearly, with time, priority areas will change and the online ‘living’ Research Agenda will evolve to reflect these changes. Indeed, although the scope of the platform is currently restricted to clinical IPD on SCH and STHs, IDDO is committed to responding to the future needs of disease control and research communities. This includes, where practicable, broadening its scope to capture new data and new diseases. For example, in response to strongyloidiasis being included under STHs in the WHO’s priority list of NTDs, future searches to identify studies with suitable IPD will be designed to capture data on treatment of infection by *Strongyloides stercoralis* ^124^. Ultimately, of course, the scope and sustainability of this platform and others are limited by resources and funding. Although the principles of data sharing are widely acclaimed and highly encouraged, stable funding streams are crucial to sustain data platforms as digital resources for the research and disease control communities for future generations. Resources to keep platforms operational, active and updated will ensure that the utility of clinical data and the beneficial impact of data-sharing are fully realised. 

## Data availability

No data are associated with this article.
