# Supplementary material for: Improving anthelmintic treatment for schistosomiasis and soil-transmitted helminthiases through sharing and reuse of individual participant data
Source: Wellcome Open Res. 2022 Jan 5;7:5. [Version 1] doi: 10.12688/wellcomeopenres.17468.1 (PMC9020536; doi:10.12688/wellcomeopenres.17468.1)
Supplement: Peer review history

# Peer review: Reviewer response for version 1

**DOI:** 10.21956/wellcomeopenres.19316.r49330

**Published:** 2022-04-19

**Authors:** Nana-Kwadwo Biritwum

**Affiliations:** 1 National Neglected Tropical Disease Control Programme, Ghana Health Services (GHS), Accra, Ghana

Improving anthelminthic treatment for schistosomiasis and soil-transmitted helminthiasis through sharing and reuse of individual participant data.

 This is an excellent article on the subject area of schistosomiasis and soil-transmitted helminthiasis control and elimination involving ensuring decisions, recommendations, and guidelines are driven by strong evidential data. The Infectious Diseases Data Observatory (IDDO) will contribute in no small way to informing the thinking around global technical and priority topics for further research and implementation under schistosomiasis and soil-transmitted helminthiasis by institutions, organizations, and partners including the WHO, and therefore the research agenda. Information from this platform will be most useful to technical advisory groups including the WHO technical advisory group on schistosomiasis and soil-transmitted helminthiasis. 

 The article clearly outlines and articulates a clear agenda and processes to enable the achievement of its goal of establishing a scientific engagement process involving a diverse group of research partners whose efforts are focused on schistosomiasis and soil-transmitted helminthiasis. The role of the WHO in providing technical leadership in working with countries should however be acknowledged somewhere in the paper. 

 The gaps and the methodology for identifying these gaps are well discussed. However, the paper makes little reference to discussions or information on the currently available schistosomiasis and soil-transmitted helminthiasis subject matter experts, research partners, observatories, data repositories, and other data platforms within this space, and what they do. This can be mentioned without going into great detail about what these platforms offer and their potential role in supporting the research agenda and IDDO. These could be identified and briefly touched on to provide more context with specific examples. The utility and potential for interactions and interoperability between IDDO and other platforms though not a key objective of this paper could serve as an interesting area to be considered. Noting that the highest burden of schistosomiasis and soil-transmitted helminthiasis occurs in Africa, the ESPEN data portal, and also Global Atlas of Helminth Infection (GAHI), and how these platforms will interact with IDDO will provide more clarity on the functionality and purpose of IDDO while dispelling any views of potential duplication. 

 The value of routine reporting data from country NTD programs in Africa in particular with the highest disease burden and research needs and the role of the Joint Application Package (JAP) for the Preventive Chemotherapy Neglected Tropical Disease (PC-NTDs) reporting including research data has not been captured in the paper. This, in my estimation, could throw some light on the gaps in available or currently used data collection tools employed by countries. 

 In conclusion, this is a brilliant paper with all the expected elements required for the establishment of the IDDO using individual participant data (IPD). The paper is written in clear technical but easy-to-understand language. Additional comments made may be considered if it is convincing enough which I will leave to the discretion of the writers and editors, however, the article should be accepted for indexing with or without addressing these comments.

Does the article adequately reference differing views and opinions?

Yes

Are all factual statements correct, and are statements and arguments made adequately supported by citations?

Yes

Is the Open Letter written in accessible language?

Yes

Where applicable, are recommendations and next steps explained clearly for others to follow?

Partly

Is the rationale for the Open Letter provided in sufficient detail?

Yes

Reviewer Expertise:

Neglected Tropical Diseases implementation research.

I confirm that I have read this submission and believe that I have an appropriate level of expertise to confirm that it is of an acceptable scientific standard.

# Peer review: Reviewer response for version 1

**DOI:** 10.21956/wellcomeopenres.19316.r47843

**Published:** 2022-01-27

**Authors:** John Gilleard

**Affiliations:** 1 Dept of Comparative Biology and Experimental Medicine, Host-Parasite Interactions Program, University of Calgary, Calgary, AB, Canada

This review describes the priority research themes of the clinical data platform of the Infectious Diseases Data Observatory. This is an extremely important initiative that aims to promote research and provide a framework for meaningful data sharing and research collaborations aiming to eliminate NTDs. The paper is extremely well written and will be a valuable resource for NTD workers.

 I have some minor comments on a number things that are missing and inclusion of which could perhaps add value.

- In the section on “Characterizing temporospatial heterogeneity” I don’t see a comment on the possibility of genetic variation of the parasite potentially being a contributory factor for this (this goes much wider than potential drug resistance mutations) , eg , cryptic species, hybrids, or within species genetic variation.
- The recent paper by Hurlimann et al Lancet Infect Dis. 2022 Jan;22(1):123-135 ^1^ is an excellent example of several key points being made and would be worth citing. It reports three well designed and controlled clinical trials showing the higher efficacy of Ivermectin and albendazole combination therapy against T. trichiura and so is very relevant in the section on “improving evidence base for treatment regimes” when combination treatments are discussed. Also the study finds markedly  lower efficacy of the combination treatment in the Cote d’Ivoire trial than in the Laos or Pemba,Tanzania trials and so is very relevant to the “Characterizing temporo-spatial heterogeneity” section - particularly as these are well matched studies in the three regions in terms of design, execution and analysis (comparisons are often  confounded by very different methodologies of different studies).

There are some important themes/potential research priorities which don’t seem to have emerged from the process of developing the research priorities through the consultative process and so are not covered in the review. It might be worth some brief comments on such aspects.

 In my opinion, three important ones are:  

1. The standardization of collecting, fixing and archiving (making biobanks) of samples associated with clinical studies and data. This is hugely important for future studies monitoring changes in parasite species over time, investigating possible reasons for differences in efficacy across studies and also investigating the potential emergence of resistance.  
2. In the priority research theme “Evaluating new antigen detection and molecular diagnostics”; drug resistance markers are not mentioned nor is investigating general  genetic differences between parasite populations that might contribute to the differing responses to drugs treatments. A comment on these would be worth including.
3. One Health aspect is not mentioned. Eg. the investigation of potential role of animal reservoirs in variability on progress in elimination programs. Again this is a  potentially important research area that doesn’t seem to have emerged from the consultative process.   

Does the article adequately reference differing views and opinions?

Yes

Are all factual statements correct, and are statements and arguments made adequately supported by citations?

Yes

Is the Open Letter written in accessible language?

Yes

Where applicable, are recommendations and next steps explained clearly for others to follow?

Yes

Is the rationale for the Open Letter provided in sufficient detail?

Yes

Reviewer Expertise:

Parasitology

I confirm that I have read this submission and believe that I have an appropriate level of expertise to confirm that it is of an acceptable scientific standard.

## References

[1] : Efficacy and safety of co-administered ivermectin and albendazole in school-aged children and adults infected with Trichuris trichiura in Côte d'Ivoire, Laos, and Pemba Island, Tanzania: a double-blind, parallel-group, phase 3, randomised controlled trial. *The Lancet Infectious Diseases* .2022;22(1) : 10.1016/S1473-3099(21)00421-7 123-135 10.1016/S1473-3099(21)00421-7 34856181
